# Uveal melanoma incidence trends in Canada: 1992–2010 vs. 2011–2017

**DOI:** 10.3389/fmed.2022.1001799

**Published:** 2023-01-24

**Authors:** Santina Conte, François Lagacé, Feras M. Ghazawi, Leila Cattelan, Siddharth Nath, Jobanpreet Dhillon, Hacene Nedjar, Elham Rahme, Denis Sasseville, Miguel N. Burnier, Ivan V. Litvinov

**Affiliations:** ^1^Faculty of Medicine and Health Sciences, McGill University, Montréal, QC, Canada; ^2^Division of Dermatology, McGill University, Montréal, QC, Canada; ^3^Division of Dermatology, University of Ottawa, Ottawa, ON, Canada; ^4^Department of Ophthalmology, McGill University, Montréal, QC, Canada; ^5^Division of Clinical Epidemiology, McGill University, Montréal, QC, Canada

**Keywords:** uveal melanoma, incidence, epidemiology, Canada, provinces

## Abstract

**Background/aims:**

Uveal melanoma is the most common type of non-cutaneous melanoma and the most common ocular malignancy in the adult population, especially affecting Caucasians (98% of cases). Despite its low incidence rate, we have noted increasing incidence trends in recent years.

**Methods:**

We analyzed uveal melanoma incidence data using the Canadian Cancer Registry (CCR) for 2011–2017 years. The data was examined using the International Classification of Diseases for Oncology, Third Edition, codes for all uveal melanoma subtypes. The data for 2011–2017 was then compared to previously published work by our research group for uveal melanoma incidence in Canada between 1992 and 2010 using the same methodology.

**Results:**

Between 2011 and 2017, 1,215 patients were diagnosed with uveal melanoma, 49% of whom were females. The percentage distribution of uveal melanoma between the sexes was similar between 1992–2010 and 2011–2017, whereby of the 2,215 diagnoses of uveal melanoma in 1992–2010, 47.9% were females. The change in the incidence rate for this cancer has doubled between 1992–2010 and 2011–2017, from 0.074 to 0.15 cases per million individuals per year. Our study documents that the Canadian 2011–2017 age-standardized incidence rate (ASIR) for uveal melanoma against the World Health Organization (WHO) 2000–2025 world population standard was 5.09 cases per million individuals per year (95% confidence interval, 4.73–5.44), as compared with the 1992–2010 rate of 3.34 cases per million individuals per year (95% confidence interval, CI 3.20 to 3.47).

**Conclusion:**

This work demonstrates an ongoing, steady increase in uveal melanoma incidence in Canada in recent years.

## Introduction

Although rare, uveal melanoma is the most common primary intraocular malignancy in adults, primarily affecting Caucasians and men over the age of 65 (1). We reported a comprehensive, population-based study of uveal melanoma and other ocular malignancies (2–6) in Canada between 1992 and 2010, in which we demonstrated that the incidence of this cancer is on the rise across the country, with a slight male predominance at that time. The most common histological subtype was malignant melanoma, not otherwise specified, and the choroid was the most frequent site involved.

## Methods

Data for this study was collected from the Canadian Cancer Registry (CCR) for 2011–2017 years and examined according to the International Classification of Diseases for Oncology, 3rd Edition (ICD-O-3) codes, for all subtypes of uveal melanoma, in a similar manner as previously reported (2–10), in accordance with the QICSS-RDC-668035 and 13-SSH-MCG-3749-S001 protocols approved by the Social Sciences and Humanities Research Council of Canada (SSHRC) and the Québec Inter-University Centre for Social Statistics (QICSS). The CCR is a population-based registry that combines data from each of the provincial and territorial cancer registries, with the exception of Québec. Provincial and territorial cancer registries collect and report all primary cancers diagnosed since 1992 to Statistics Canada, which maintains the CCR (11). The CCR meets the reporting standards of the International Agency for Research on Cancer (IARC). Incidence data from the CCR included all Canadian provinces and territories, with the exception of Québec, for which data was not available from Le Régistre Québecois du Cancer (LRQC). Information gathered from the CCR included sex, year of diagnosis, age at diagnosis, morphology, topography and geographic location/residence of patients. Data was extracted from the CCR using SAS statistical software at a QICSS research laboratory and vetted according to Statistics Canada standards. All frequency counts less than 5 and cases arising from populations <5,000 must be omitted, and all values extracted from the lab must be rounded to the nearest multiple of 5. Two types of incidence rates were calculated: crude incidence rates and age-standardized incidence rates (ASIR) for Canada against the World Health Organization (WHO) 2000–2025 world population standard. A crude incidence rate is the number of new uveal melanoma diagnoses per 1,000,000 individuals in the population per year. ASIR are a weighted average, based on the WHO 2000–2025 world population standard, of the number of new cancer cases per 1,000,000 individuals in a 5-year age group diagnosed during a year, divided by the total number of people in that age group that year (12). The goal of using ASIR is to calculate the incidence rate that would occur if the population of interest had the same age distribution as the given standard population, standardizing rates across varying populations (12). For simplicity, crude incidence rates are referred to as incidence rates throughout this manuscript. Elements such as clinical disease stage and patient ethnicity were not available from this registry. Subsequently, we analyzed both clinical and pathological characteristics, such as morphology, topography, age, sex and geographical location of patients over a 7-year time period, and compared this data to the findings for 1992–2010. This study uses the same methodology, including that of data collection and analysis, as our previous epidemiological study of uveal melanoma in Canada (5).

## Results

**FIGURE 1:**
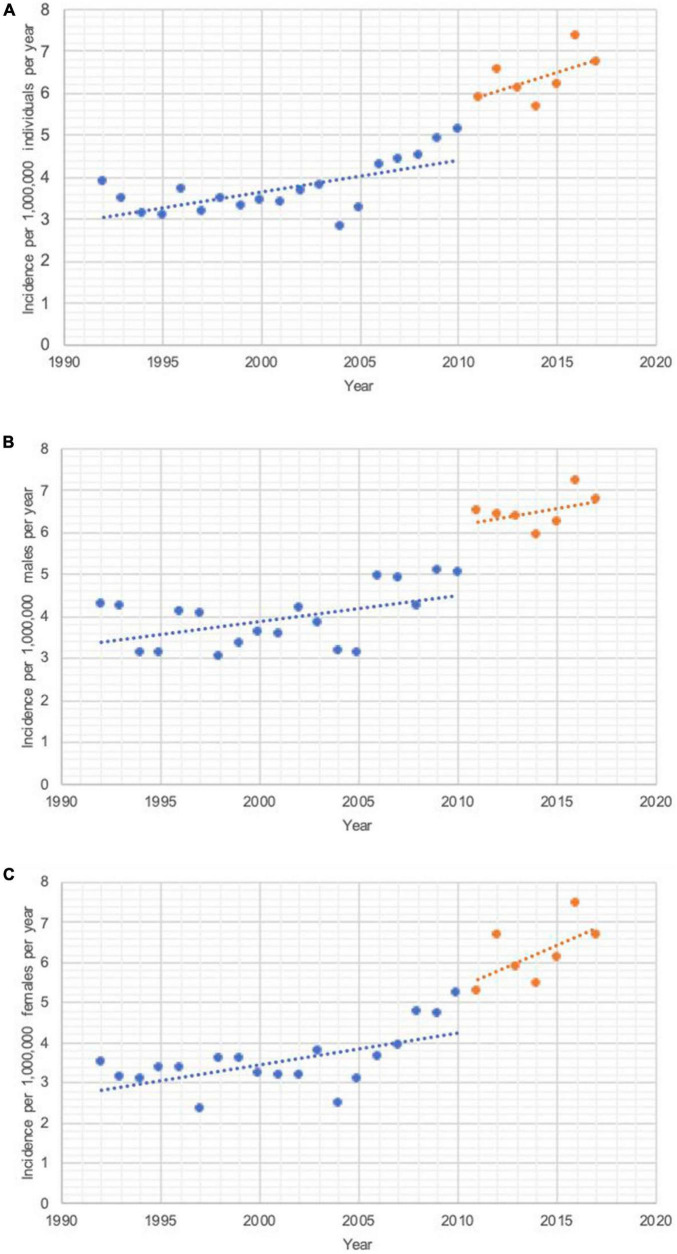
Incidence rates (cases per million individuals per year) during 2011–2017 vs. 1992–2010 years. **(A)** All uveal melanoma cases. A total of 2011–2017: (*R*^2^) = 0.32; *p* = 0.19 and the slope of the line was 0.15 ± 0.097 cases per million individuals per year. The average incidence rate of uveal melanoma in Canada was 6.36 cases per million individuals per year. A total of 1992–2010: (*R*^2^) = 0.42; *p* = 0.003 and the slope of the line was 0.074 ± 0.021 cases per million individuals per year. The average incidence rate of uveal melanoma in Canada was 3.75 cases per million individuals per year. **(B)** Uveal melanoma in males. A total of 2011–2017: (*R*^2^) = 0.17; *p* = 0.35 and the slope of the line was 0.081 ± 0.079 cases per million males per year. The average incidence rate of uveal melanoma in males in Canada was 6.50 cases per million individuals per year. A total of 1992–2010: (*R*^2^) = 0.30; *p* = 0.016 and the slope of the line was 0.069 ± 0.026 cases per million males per year. The average incidence rate of uveal melanoma in males in Canada was 3.93 cases per million individuals per year. **(C)** Uveal melanoma in females. A total of 2011–2017: (*R*^2^) = 0.36; *p* = 0.16 and the slope of the line was 0.21 ± 0.13 cases per million females per year. The average incidence rate of uveal melanoma in females in Canada was 6.22 cases per million individuals per year. A total of 1992–2010: (*R*^2^) = 0.36; *p* = 0.007 and the slope of the line was 0.079 ± 0.025 cases per million females per year. The average incidence rate of uveal melanoma in females in Canada was 3.54 cases per million individuals per year.

In total, 1,215 patients were diagnosed with uveal melanoma during 2011–2017, 49% of which were females. The following ICD codes were considered: C69.3 (choroid) and C69.4 (ciliary body, including iris). Data for ICD code C69.2 (retina) was also reviewed in case the tumors were inappropriately classified as arising in the retina. The uvea is a composite of the choroid, ciliary body and iris. However, the retina was included as a site for this type of intraocular cancer because at times, choroidal melanomas push against the retina causing retinal detachment. Therefore, the choroidal mass may also appear as a mass deep to the retina, potentially resulting in misclassification (13). The sum of these sites provided us with the total number of uveal melanomas in Canada during this period. There were 1,085 cases (89.3%) at the level of the choroid and 130 (10.7%) in the ciliary body/iris. There were less than 5 uveal melanoma cases of the retina (i.e., misclassification), which were not extractable due to restrictions imposed by Statistics Canada to maintain patient confidentiality. On the national scale, the incidence of this cancer has continued to increase in recent years, with an annual rate of increase doubling from 0.074 ± 0.021 (during 1992–2010, IQR 45–85 cases per year, median 60 cases per year) to 0.15 ± 0.097 (during 2011–2017, IQR 155–205 cases per year, median 170 cases per year) cases per million individuals per year (*p* < 0.001) (Figure 1A). The mean annual incidence rate also increased from 3.75 (1992–2010) (5) to 6.36 (2011–2017) cases per million individuals per year (Figure 1A).

This malignancy had a comparable incidence in males (6.50 cases per million individuals per year) and females (6.22 cases per million individuals per year) (*p* = 0.8) (males IQR 80–100 and median 85, females IQR 70–105 and median 85 cases per year), with a corresponding incidence rate ratio (IRR) of 1.04 for the time period (1992–2010: males = 3.93, females = 3.54, IRR = 1.11) (Figures 1B, C). Such a decrease in the IRR indicates that the incidence of this malignancy is increasing slightly more rapidly in females than it is in males. This was confirmed by analyzing each sex’s respective slopes, whereby the incidence rate has increased more rapidly in females than it has in males, with respective rates of change of 0.21 ± 0.13 (females) and 0.081 ± 0.079 (males) cases per million individuals per year (1992–2010: females = 0.079 ± 0.025, males = 0.069 ± 0.026) (Figures 1B, C).

Figure 1 enables us to compare how uveal melanoma incidence rates evolve and differ not only between sexes, but also between the compared time periods. A thorough description of the angular coefficients measuring the slope of each segment of both sexes and time periods can be found in the legend of Figure 1. Of note, while there is a well-defined change in slope when analyzing both sexes together or females alone, with the rate of change doubling for both sexes combined (0.15 from 0.076) and tripling for this change for females was observed (0.21 from 0.079). This change is less pronounced in males, where we observe a more subtle increase in the rate of incidence change for this malignancy (0.081 from 0.062).

The mean age of diagnosis was 62.6 ± 13.7 years for males and 61.0 ± 15.4 years for females (61.8 ± 14.6 during 2011–2017 vs. 61.49 ± 14.21 years in 1992–2010 for both sexes) (Table 1). Age range analysis confirmed these findings and showed that 57.2% of patients with uveal melanoma during 2011–2017 were ≥60 (1992–2010: 58.2%) (Table 1). A minority of patients were <40 years old in both time periods (2011–2017: 7.4%, 1992–2010: 7.2%) (Table 1).

**TABLE 1:** Demographic characteristics of patients with uveal melanoma in Canada diagnosed between 2011 and 2017.

	No of patients*	% of total cases
**Histological subtypes**		
Malignant melanoma, not otherwise specified (NOS)	1,205	99.59
Amelanotic melanoma	5	0.41
Total uveal melanoma	1,210	100.00
**Sex**		
Male	615	50.83
Female	595	49.17
**Age**		
0–19	5	0.41
20–39	85	7.00
40–59	430	35.39
60–79	560	46.09
80 +	135	11.11
Mean age at diagnosis for uveal melanoma	61.83 (SD 14.61)	
Mean age at diagnosis for uveal melanoma–males	62.61 (SD 13.73)	
Mean age at diagnosis for uveal melanoma–females	61.03 (SD 15.43)	
**Site description**		
**Classified as being in the retina**	**<5, data not extractable**	**<5, data not extractable**
Choroid	1,085	89.30
Ciliary body and iris	130	10.70

*Rounded (up or down) to a multiple of 5. We acknowledge that classification of tumor being in the retina by the CCR (<5 cases) is inappropriate considering that the tumor is in the choroid pushing against the retina.

Similar trends were also noted with regards to the anatomic location of uveal melanoma during 2011–2017 as compared to 1992–2010, whereby the majority of cases occurred in the choroid (2011–2017: 89.3%, 1992–2010: 84.2%). Otherwise, the ciliary body (eyeball, iris, lens, sclera and uveal tract) continues to be the second most common site (2011–2017: 10.7%, 1992–2010: 14.7%). We observe a relative increase in the number of malignancies in the choroid as compared to the ciliary body recently (Table 1). Regional trends for uveal melanoma in Canada are presented in Table 2.

**TABLE 2:** Comparison of uveal melanoma incidence rates across Canadian provinces.

Province	Cases*	Population	Incidence per 1,000,000 individuals-year	Lower CI (95%)	Upper CI (95%)
Atlantic provinces (Newfoundland and Labrador, Prince Edward Island, Nova Scotia, and New Brunswick)	45	2,375,507	2.71	1.97	3.62
Ontario	690	13,633,639	7.23	6.70	7.79
Manitoba	25	1,281,235	2.79	1.80	4.11
Saskatchewan	30	1,110,054	3.86	2.60	5.51
Alberta	220	4,044,619	7.77	6.78	8.87
British Columbia	200	4,709,418	6.07	5.26	6.97
Canada (excluding Québec)	1,210	27,271,765	6.34	5.99	6.71

Due to small numbers, Newfoundland and Labrador, Prince Edward Island, Nova Scotia, and New Brunswick were calculated as a single entity: Atlantic provinces. Data was not available for the northern territories due to low cumulative frequency counts. Data was not available for the Province of Québec due to errors in data collection at the level of the *Régistre Québecois du Cancer*. *Rounded (up or down) to a multiple of 5.

The ASIR for uveal melanoma against the WHO 2000–2025 world population standard in Canada for 2011–2017 years was 5.09 cases per million individuals per year (95% confidence interval, 4.73–5.44), as compared with the 1992–2010 rate of 3.34 cases per million individuals per year (95% confidence interval, CI 3.20 to 3.47), demonstrating an ongoing, steady increase in incidence amongst Canadians in recent years.

## Discussion

This study presents current trends on Canadian uveal melanoma incidence during 2011–2017 years and compares this data to the 1992–2010 time period. The overall incidence of uveal melanoma in Canada was 6.36 cases per million individuals per year (6.50 in males and 6.22 in females), up from 3.75 cases per million individuals per year in 1992–2010 (3.93 in males and 3.54 in females). In comparison, the incidence of uveal melanoma in the United States between 2010 and 2015 was 4.64 cases per million individuals per year (11). To our knowledge, no other groups have studied the epidemiology of uveal melanoma in their population in the last decade.

Primarily found in the Caucasian population, uveal melanomas differ from cutaneous melanomas such that ultraviolet radiation has not been definitively proven to increase the risk of this cancer, although occupational exposure to ultraviolet light through welding is a well-documented risk factor (14, 15). Other risk factors for this cancer include fair skin, light eye color, inability to tan, ocular or oculodermal melanocytosis, iris/choroidal nevus and BRCA1-associated protein 1 (BAP1) mutations (1). In this study, we noted a continuity of trends between 1992–2010 and 2011–2017 documenting the increase in uveal melanoma incidence in Canada, which is also indicated by the increase in the ASIR from 3.34 during 1992–2010 to 5.09 during 2011–2017 years. Similarly, an increase in incidence of cutaneous melanoma was observed in multiple Canadian studies (8–10, 16). When compared to global trends, the mean age-adjusted incidence rate of uveal melanoma in Canada is comparable to the earlier documented worldwide rate of 4.3 cases per million individuals per year (17).

Epidemiological trends of this intraocular malignancy vary from one country to another, which may be attributed to varying risk factors between populations. For instance, in a study conducted by Park et al. the ASIR for this cancer was found to be relatively lower in South Korean than in Caucasian individuals (18), despite an increase in its incidence rate in recent years. In comparison, the ASIR in Republic of Korea between 1999 and 2011 was 0.42 per million individuals per year, while it was found to be 3.34 (1992–2010) and 5.09 (2011–2017) in our current and our prior Canadian studies (5). Similarly, a 2011–2013 study conducted in Japan found the annual incidence rate of this cancer to be 0.64 cases per million (19), while a study in China reported 0.6 cases per million per year between 1990 and 2005 (19). In contrast, epidemiological analyses have noted similar incidence trends for this cancer in the United States, with a Surveillance, Epidemiology and End Results (SEER) study conducted between 1973 and 2013 finding that the mean age-adjusted annual incidence of uveal melanoma was 5.2 cases per million individuals (20), and another conducted between 2010 and 2015 concluding that the overall incidence of this cancer was 4.64 cases per million individuals per year (21). Similarly, a study conducted in Israel between 1988 and 2007 found that the annual incidence of this cancer was 6.71 cases per million individuals per year, in line with the North American findings (19, 22). Moreover, in Australia individuals are facing similar rates of this cancer, with 7.6 cases per million individuals per year being diagnosed (23). Comparable trends were observed throughout Europe, with the incidence rate of uveal melanoma in the Swedish population ranging between 6.5 and 11.6 cases per million individuals per year between 1960 and 2010 (24), 9.5 uveal melanoma cases per million individuals per year being diagnosed in Ireland between 2010 and 2015 (25), and 6.41 persons per million being diagnosed with uveal melanoma in Germany between 2009 and 2015 (26).

Overall, it appears that the incidence rate for this cancer is higher in Europe, Australia, and North America, as compared to Asia (19). We hypothesize that these significant differences are due to disparities in the prevalence of risk factors for this malignancy in different populations. For instance, in countries with high proportion of individuals with fair skin and lighter eye color, such as the United States, Canada, Australia, Sweden, Ireland, and Germany, we note a substantially higher incidence of uveal melanoma, whereas other studies have established that darker eye color is protective against uveal melanoma (27–29). Moreover, a case-control study assessing the host factors and risks of uveal melanoma found a relative risk (RR) of 6.5 for those with an ancestry from northern latitudes, especially those with Northern European ancestry, and an RR of 3.8 for light skin color as compared with dark skin tones (30). Altogether, these findings may allow us to better understand the epidemiological picture of uveal melanoma in Canada. Seeing Canada as a cultural mosaic, it can be deduced that UM rates vary between geographical regions due to differences in the populations that are settled there.

Due to the nature of large, population-based studies, this retrospective study had several limitations including missing data and a risk of misclassification of patients. An important limitation was the lack of data for the province of Québec during this period. Additionally, the CCR did not contain data on patients’ ethnicity, disease stage or other clinical parameters. Moreover, low frequency counts limited our ability to conduct additional detailed analyses by sex, by age and by jurisdiction. Small frequency counts across provinces forced us to group jurisdictions to comply with confidentiality guidelines imposed by Statistics Canada. Because the CCR is a dynamic database that includes data collected by each province or territory’s own cancer registry, it is affected by delays or errors in reporting at the provincial level, which may have led to under-detection of this cancer by the CCR and impacted our findings. Under-coverage of the incidence of a cancer may be due to provincial or territorial cancer registries not using death certificates as a source of identifying cancer tumors, different definitions of what constitutes a reportable or malignant tumor, difficulty in diagnosing certain types of tumors due to their location in the body, differences in coding practices, data entry or processing between the provinces/territories, or failure to report tumors diagnosed and treated outside of an individual’s place of residence (31). Finally, in conducting an epidemiological analysis of cancer trends, it is important to understand how the use of capture rates may have caused an apparent increase in incidence. This may be due to the improved diagnostic capability of ophthalmic ultrasound in recent years, which were initially less frequently used due to their low resolution (14, 32), widespread introduction of diabetic retinopathy screening, or increasing frequency of cataract surgery (24), among other reasons. A last limitation to this study is that we cannot ascertain whether the excess of UM cases as compared to previous decades is due to smaller UM tumors treated conservatively as opposed to large UM tumors with unspecified topography, because such data, unfortunately, is not recorded by the CCR.

## Conclusion

Overall, we note a continuity of uveal melanoma increasing incidence trends in Canada in recent years. Such findings are alarming because despite the fact that this cancer is relatively rare, up to 50% of patients are noted to have metastatic disease and experience high morbidity and mortality (33). Hence, concerted efforts are needed to increase disease awareness and advise the public to protect their eyes from harmful UV radiation exposure. Finally, additional research is needed to better understand the driving forces behind this significant increase in uveal melanoma cases across the country in recent decades, whether due to individual or environmental factors, or better awareness and diagnostic ability by patients and physicians.

## Data availability statement

The original contributions presented in this study are included in the article/supplementary material, further inquiries can be directed to the corresponding authors.

## Ethics statement

This presented study was conducted in accordance with protocols approved by the Social Sciences and Humanities Research Council of Canada (SSHRC) and the Québec Inter-University Centre for Social Statistics (QICSS), respectively, protocol numbers: CISS-RDC-668035 and 13-SSH-MCG-3749-S001. Further, in accordance with the institutional policy, this study received an exemption from the McGill University Research Ethics Board review.

## Author contributions

SC, FG, FL, LC, and IL collected, plotted and analyzed the data, prepared the figure, wrote the manuscript, and prepared the revised version of the manuscript. SN, JD, DS, and MB analyzed and interpreted the data and co-wrote the manuscript. HN and ER performed the statistical analyses. MB, DS, and IL designed and supervised the study. All authors contributed to the article and approved the submitted version.
